# Interfacial properties of the ionic liquid [bmim][triflate] over a wide range of temperatures

**DOI:** 10.1039/c8ra00915e

**Published:** 2018-03-13

**Authors:** José L. Rivera, Luis Molina-Rodríguez, Mariana Ramos-Estrada, Pedro Navarro-Santos, Enrique Lima

**Affiliations:** Graduate School of Physics Engineering, Universidad Michoacana de San Nicolás de Hidalgo Morelia C.P. 58000 Michoacán Mexico jlrivera@umich.mx; Faculty of Chemical Engineering, Universidad Michoacana de San Nicolás de Hidalgo Morelia C.P. 58000 Michoacán Mexico; Institute of Chemical Biology Sciences, Universidad Michoacana de San Nicolás de Hidalgo Morelia C.P. 58000 Michoacán Mexico; Laboratorio de Fisicoquímica y Reactividad de Superficies (LaFReS), Instituto de Investigaciones en Materiales, Universidad Nacional Autónoma de México Circuito Exterior S/N, CU, Del. Coyoacán Ciudad de México Mexico

## Abstract

We carried out molecular dynamics simulations of the liquid/vacuum equilibrium of the ionic liquid [bmim][triflate] in a wide range of temperatures (323.15 to 573.15 K). The results showed liquid phases with high densities even at temperatures close to the decomposition temperature of the liquid. The density and surface tension behaviors are linear across this wide range of temperatures, which is an extension of the behaviors of these systems at low temperatures, where these properties have been experimentally measured. The interfacial region shows peaks of adsorption of the ions; they are ordered, with the alkyl chains of the [bmim] cations pointing out of the liquid, and the tailing angle of the chains becomes 90° at higher temperatures. The alkyl chains are part of the outermost interfacial region, where intra- and intermolecular tangential forces are in equilibrium; thus, they do not contribute to the total surface tension. Unlike simpler organic liquids, the surface tension is composed of positive normal contributions of intermolecular interactions; these are almost in equilibrium with the negative normal contributions of intramolecular interactions, which are mainly vibrations of the distance and the angle of valence. The pressure profiles show that the molecules are in ‘crushed’ conformations internally in the bulk liquid and even more so in the normal direction at the interface. The total pressure profiles show values with physical meaning, where the tangential peaks show higher values than normal pressures and give rise to the surface tension. Short cutoff radii for the calculation of intermolecular forces (less than 16.5 Å) produce a system that is not mechanically stable in the region of the bulk liquid (confirmed by radial distribution function calculations); this produces a difference between the normal pressure and the average of the tangential pressures, which affects the calculation of the surface tension due to overestimation by up to 20% when using the global expression, which is extensively used for the calculation of surface tension. The use of a sufficiently long cutoff radius avoids these mechanical balance problems.

## Abstract 2

Picture of the ionic liquid [bmim][triflate] at the vapor/vacuum interface. The liquid density and surface tension show linear behavior in a wide temperature range. The outer interfacial space shows a zero-surface tension zone.

## Graphical abstract

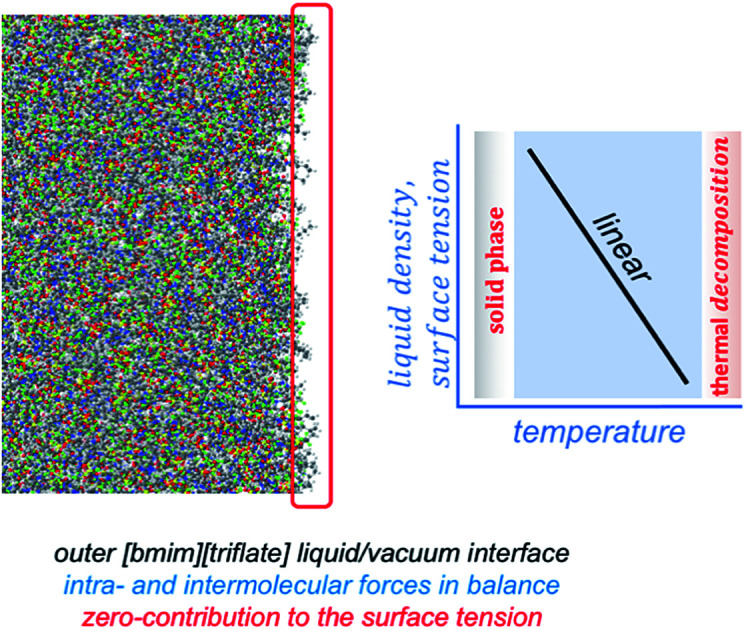

## Introduction

Ionic liquids combine relatively large organic cations and organic and inorganic anions; this prevents the formation of ordered crystals over a wide range of temperatures, including room temperature. These liquids are more complex than ordinary organic liquids, and it is thought that they can be best described as fluids that self-assemble into amphiphilic nanostructures.^1–3^ Simulations of molecular dynamics in the liquid/vacuum equilibrium using 368 pairs of ions with a simulation area of ∼20 nm^2^ and 5 ns for sampling indicate the presence of amphiphilic interfaces. This is due to ordering in the interfaces because the alkyl chains of diverse ionic liquids based on imidazole leave the bulk liquid, pointing towards the vacuum; this is similar to the behavior of complexes that form water bridges with amphiphilic molecules.^4^ Other simulations, with smaller samples (216 pairs of ions), a smaller surface area (∼17 nm^2^), and 2 ns for sampling, produce similar results.^5^

On the other hand, the thermophysical properties of ionic liquids are unique. Their vapor pressure is extremely low, and the dependence of their surface tension, *γ*, on temperature is different from that of most organic liquids (*γ*∝*T*^11/9^). Ionic liquids behave almost linearly with respect to temperature; therefore, the linear van der Waals–Guggenheim equation, which describes the behavior of molten metals, also accurately describes the behavior of many ionic liquids.^6^ Some exceptions to this linear behavior have been attributed to problems arising during the preparation of the liquids, such as insufficient purification.^6,7^ The behavior of *γ* with respect to the temperature of the ionic liquid [bmim][triflate] has been studied experimentally by Součková *et al.* in the temperature range of 292.16 to 356.00 K,^8^ by Freire *et al.* in the range of 292.20 to 343.20 K,^9^ and by Cione *et al.* at 298.15 K.^10^ All the reported sets of data show linear behavior. Freire *et al.*^9^ used force measurements (the du Noüy ring method) to obtain their data, and the purity of the employed mixture was >98%. Meanwhile, Součková *et al.*^8^ also used force measurements; however, they employed two methods (the du Noüy ring and Wilhelmy plate methods). They reported that the data acquired from the du Noüy ring method showed seven times more scatter than those from the Wilhelmy plate method; the purity of their mixture was >99.5%.

The density of ionic liquids, *ρ*_L_, unlike many organic liquids, also presents linear behavior with respect to temperature. The behavior of *ρ*_L_ with respect to the temperature of the ionic liquid [bmim][triflate] has been studied experimentally by several groups, and data have been reported for a temperature range of 288.15 to 343.15 K at ambient pressure.^11–14^ The effects of pressure on *ρ*_L_ have been studied by Gardas *et al.*^15^ They found that, as with many common liquids, there is a weak dependence of *ρ*_L_ on pressure; thus, when the pressure changes from 0.10 to 10.0 MPa, the density of the ionic liquid only increases by 0.56% at 373.15 K.

Many measurements of *ρ*_L_ and *γ* have only been studied over a small range of temperatures and pressures; therefore, more experimental and simulation measurements are necessary in order to understand the behavior of these fluids over a wide range of temperatures and pressures. It is also necessary to obtain more information on how the arrangement of the alkyl chains of the [bmim] cations contributes to the interface, and their possible effects on the interfacial properties.

In this paper, we report *ρ*_L_ and *γ* in the liquid/vacuum equilibrium region for a wide range of temperatures, from 323.15 to 573.15 K, which approach the thermal stability limit. We also explain how the molecules are organized at the interface, as well as how the components of the pressure tensor affect the surface tension.

The methods and parameters of the interactions used in this work are described in the Methodology section. In the Results section, we discuss the main results, in terms of the total density profiles, by component; the profiles of the concentration of sites in the interface and their structuring and ordering; and the profiles of pressure and surface tension. We also present an analysis of the main contributors to the surface tension. The Conclusions section summarizes our findings.

## Methodology

The liquid/vacuum interface of the ionic liquid [bmim][triflate] was studied directly through the simulation of a thin layer of liquid (thickness of more than 100 Å) surrounded by a vacuum^16–18^ using the molecular dynamics method at various temperatures of between 323.15 and 573.15 K. The simulation cell consisted of a parallelepiped with dimensions of 94 × 94 × 180 Å and an interfacial area of 88.36 nm^2^ which contained 2592 pairs of ions (85 536 atoms). The initial system consisted of a cubic cell, to simulate a single liquid phase, which contained half of the ion pairs under the conditions of 298.15 K and 0.1 MPa once equilibrium was reached. This cell was repeated once in the inhomogeneous direction and was left to balance. Finally, vacuum was added in the inhomogeneous direction, and the portions of the molecules that were not entirely located at the interfaces due to the initial periodic conditions shifted. The system was initially equilibrated at 573.15 K in order to avoid the difficulty of structuring of the liquid phases at low temperatures, which requires a long simulation time to equilibrate.^19,20^ Systems at lower temperatures were obtained by cooling the system at a speed of 10 K ns^−1^. The system was simulated using the NVT assembly (number of atoms, volume, and temperature constants) using the Nosé thermostat,^21^ implemented in the open source for large-scale atomic/molecular massively parallel simulator (LAMMPS),^22^ with a time step of 1 fs.

The components of the ionic liquid were simulated using the models of the ions [bmim] and [triflate] developed by Cadena *et al.*^23^ and Lopes *et al.*,^24^ respectively. The molecular ion models are flexible and include intramolecular interactions due to vibrations of the bonding distance, valence, dihedrals, and improper angles. The intermolecular interactions of the original model of the [bmim] cation include truncated Lennard-Jones and shifted potential through a switching function, with an internal cutoff radius *r*_ci_ and an external cutoff radius *r*_ce_ of 10.5 and 12 Å, respectively. The original model of the [triflate] anion includes the Lennard-Jones truncated potential, with cutoff radii of 10 and 12 Å depending on the state of the system; long-range corrections were applied. The potentials from these two ions have been optimized in ionic liquids with other ions, but not for the [bmim][triflate] liquid; however, no other specific potential for this liquid has been reported in the literature, so we used these potentials as the best available approximation. Cione *et al.* also used this potential approximation to study how [bmim][triflate] ionic liquid wetted and deposited on silica coated with alkyl chains.^10^ In this study, we used the truncated and displaced potential without long-range corrections of the Lennard-Jones forces to ensure the compatibility of the two models. We studied the effects of *r*_ci_ on the calculated properties, using values for *r*_ci_ of 10.5, 12.5, …, 30.5 Å, while *r*_ce_ = *r*_ci_ + 1.5 Å. The maximum cutoff radius employed (2 × 32 Å), plus 10% for the neighbor list (64.0 + 6.4 Å), is smaller than the length of the simulation cell in the tangential direction, which allowed us to correctly sample the intermolecular interactions. The use of a large cutoff radius requires the computation of larger numbers of intermolecular interactions, an estimation which can be obtained by calculating the relationship between the volumes of spheres at different *r*_ce_ values, 
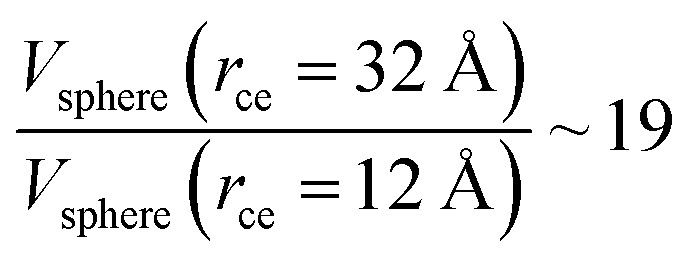
, which are directly related to the number of particles and, ultimately, to the number of intermolecular interactions (assuming no intramolecular interactions). For a shorter cutoff radius, the computational effort is considerably lower: 
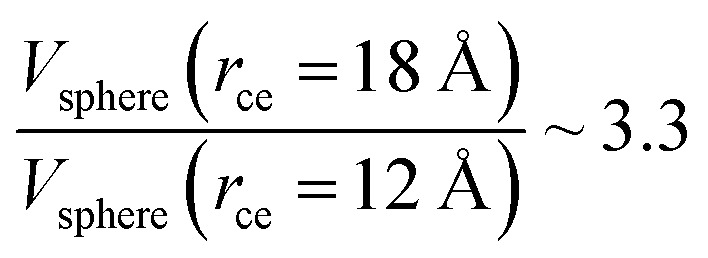
. The electrostatic interactions were modeled using the 3-D particle–particle, particle–mesh method,^25^ using the *r*_ce_ of the Lennard-Jones potential. In the inhomogeneous direction, sufficient space was added to avoid interactions between periodic liquid layers.

The pressure profiles were calculated with the tension tensor that was used to calculate the pressure profiles of Harasima,^26^ implemented in LAMMPS,^27^ where contributions to the profiles are distributed only in the two slabs that originate the interactions and not uniformly throughout all the slabs between those two slabs.^28,29^ The previous procedure were applied to all intra- and intermolecular interactions. The normal pressure profile obtained from this definition was not uniform along the interface, which was thought to make no physical sense, even though it is not experimentally feasible to validate the measurements of the components of the pressure at the interface. Despite this, the pressure profile of Harasima^26^ has been used in several studies of surface tension because when it is integrated to obtain the profile of the surface tension, the same result is obtained whether the interaction forces are distributed uniformly in several slabs or in only two slabs.^30–33^ As contributions to the Harasima^26^ pressure profiles are specifically located, regions that contain inhomogeneities in their density profiles are highlighted in the pressure profiles. *γ* was calculated through its mechanical definition:^29,34^1
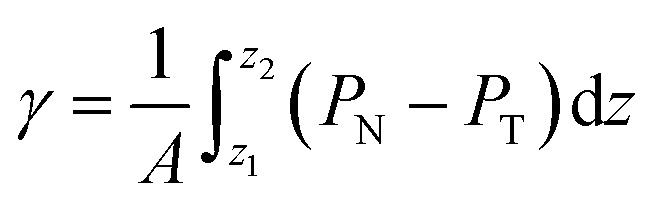
where *A* is the surface area, and *z*_1_ and *z*_2_ are the integration limits that, if they cover the entire simulation cell, require *γ* to be divided by two because there are two interfaces in the system. *P*_N_ and *P*_T_ are the normal and tangential pressures, respectively, and *z* represents the inhomogeneous direction of the simulation cell.

## Results

### Density profiles

**Fig. 1:**
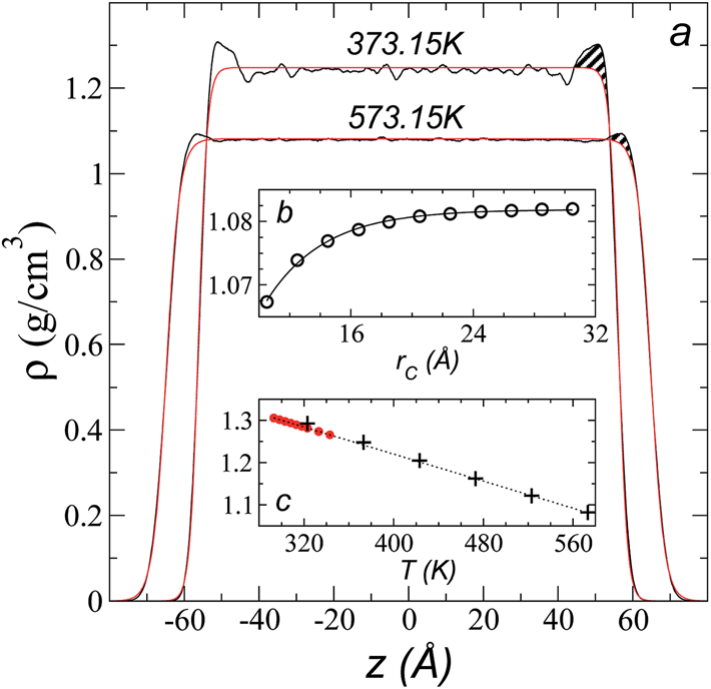
(a) Total density profiles as a function of the position on the inhomogeneous axis of the ionic liquid [bmim][triflate]. Black lines represent the results of the simulation; red lines represent the results of the adjustment to eqn (2). (b) Total density of the liquid as a function of the cutoff radius. The solid line represents the best fit to an exponential decay function. (c) Total density of the liquid as a function of temperature. Red dots represent experimental values;^14^ crosses represent the results of this work (errors calculated as the standard deviation are smaller than the symbols). The dashed line represents values of the empirical correlation of Tokuda *et al.*^14^ The *y*-axes of the three graphs represent density in the same units.

Simulations of the molecular dynamics of the ionic liquid [bmim][triflate] in an inhomogeneous arrangement in order to study the liquid/vacuum equilibrium produced the density profiles shown in Fig. 1a. The profiles show the formation of a liquid layer and its interface, with thicknesses of 112 Å (373.15 K) and 129 Å (573.15 K). The profiles show liquid regions in the bulk that range between 90 (373.15 K) and 106 Å (573.15 K), with adsorption peaks in the liquid/vacuum interface that are commonly observed in binary systems.^35,36^ These are highlighted in Fig. 1a as shaded areas above the adjustment to the next equation, which commonly describes simple systems with a single component or binary and ideal systems where there are no molecules in the vapor phase and where the density at the interface decays monotonically:^20,29,34^2
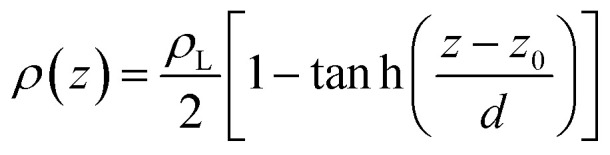
where *z*_0_ is the position of the Gibbs dividing surface and *d* is the thickness of the interface divided by 2.7192.^17^ In the obtained density profiles, none of the ions formed a vapor phase at any time during the simulation (∼60 ns). If we consider that both ions are present in the liquid/vacuum interface (which is shown below), we find that, when integrating the density profiles on the adsorption regions shown in Fig. 1a, a pair of [bmim][triflate] ions is present every 12 (373.15 K) or 34 nm^2^ (573.15 K). These additional concentrations of ions at the interface demonstrate how different these systems are from ideal systems, where the concentration drops monotonically at the interface. An analysis of the dependence of the saturated *ρ*_L_ in bulk with respect to the *r*_ci_ used in the simulation shows that *ρ*_L_ varies slightly with *r*_ci_ (Fig. 1b), presenting an underestimation of ∼1.4% at *r*_ci_ = 10.5 Å when compared with the calculated value using *r*_ci_ = 30.5 Å. This is 7.5 times higher than the largest Lennard-Jones parameter (among all the values used) associated with the equilibrium separation between two Lennard-Jones sites. In previous studies, it has been found that this *r*_ci_ is long enough to reproduce the interactions using the total Lennard-Jones potential.^37^

A graph illustrating the results of saturated *ρ*_L_ in bulk over a temperature range of 323.15 to 573.15 K using *r*_ci_ = 30.5 Å is shown in Fig. 1c. The simulation results of this work over a wide range of temperatures show a linear dependence of *ρ*_L_ on temperature; the same trend occurred in the reduced temperature range that was studied experimentally by Tokuda *et al.*^14^ (287.15 to 313.15 K). Linear extrapolations of the experimental data of Tokuda *et al.*^14^ coincide very well with the results of this work; at 573.15 K, the simulation overestimates the correlation density data by only 0.05%. The linearity of *ρ*_L_ over the wide range of temperatures studied, which reached 40 K below the thermal stability limit (613.15 K),^12,38^ suggests that these temperatures are very far from the critical temperature and that they do not influence the rapid change in saturated *ρ*_L_ with temperature.^29^ This effect is similar to that observed for the linearity of saturated *ρ*_L_ in long linear alkanes, such as *n*-hexadecane.^39–41^ Even so, we do not rule out additional effects, such as the nano-structuring of the system, which induces effects such as an unusual linear dependence of the viscosity on temperature in mixtures with water.^42^

**Fig. 2:**
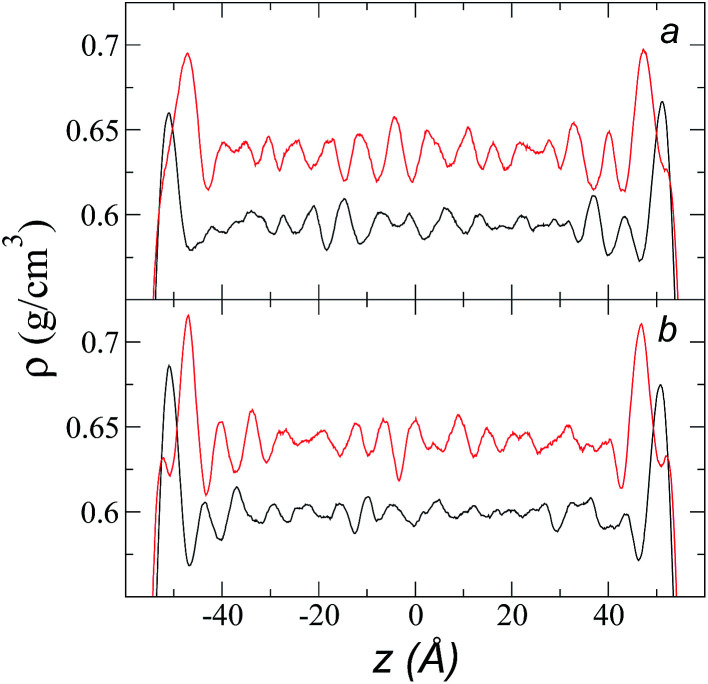
Density profiles for each of the components of the ionic liquid [bmim][triflate] at a temperature of 373.15 K and at *r*_ci_ values of (a) 10.5 Å and (b) 30.5 Å. Black and red lines represent the [bmim] and [triflate] ions, respectively.

The individual density profiles of each of the ions showed ordering at the interface and, to a lesser extent, in the bulk liquid, which did not disappear when we increased *r*_ci_ (Fig. 2). This ordering has previously been observed in simulations of ionic liquids of [bmim] and other anions;^4^ it has also been experimentally reported in X-ray reflectivity measurements, from which the formation of intercalated ion layers has been inferred.^43^ We did find when increasing *r*_ci_ that the peaks of adsorption at the interface were more pronounced with increasing *r*_ci_, which is probably due to an increase in cohesiveness because more intermolecular forces are taken into account in the Lennard-Jones potential.

### Structuration at the interface

**Fig. 3:**
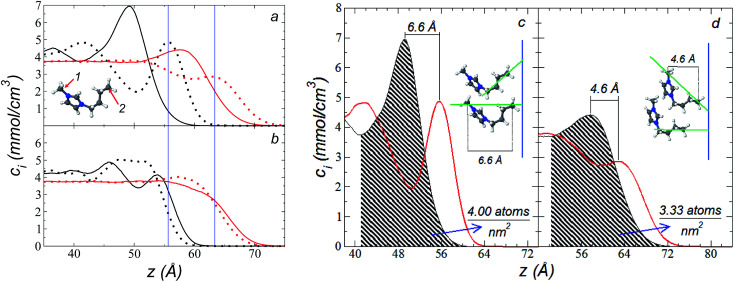
(a) Atomic concentration profiles of the carbons at the ends of the [bmim] ion, C_1_ and C_2_ (see figure), as a function of their positions on the inhomogeneous axis. Black and red lines represent the values at 373.15 and 575.15 K, respectively. Solid lines represent the C_1_ profile; dotted lines represent the C_2_ profile. Blue lines represent the values of the outer peaks of site C_2_. (b) Atomic concentration profiles of the C and S atoms of the [triflate] ion (F_3_C–SO_3_)–. Solid lines represent the profile of C; dotted lines represent the profile of S. (c and d) Expanded views of the atomic profiles of the [bmim] cation at 373.15 and 575.15 K, respectively. Black and red lines represent the C_1_ and C_2_ carbons, respectively. Blue lines represent the position of the liquid/vacuum interface. The orientations of the [bmim] cations with respect to the interface are also depicted in the figures.

The atomic concentration profiles of key sites in the structures of the ions are shown in Fig. 3a and b at temperatures of 373.15 and 573.15 K. For the [bmim] ion, the profiles of the carbons corresponding to the methyl attached to the cycle (C_1_) and the terminal carbon of the alkyl chain (C_2_), are plotted, while for the [triflate] ion, the profiles of the central atoms, C and S, are plotted. The profiles show a pronounced peak of adsorption at the interface for the C_1_ carbons of [bmim] at 373.15 K, surrounded by two adsorption peaks in the profile of the C_2_ carbons. At 573.15 K, the magnitude of the peak of the C_1_ carbons is reduced, and the inner peak of the C_2_ carbons disappears. The same occurs with the [triflate] ion at 373.15 K, where a broad adsorption peak, representing the S atoms, is surrounded by adsorption peaks of the C atoms; meanwhile, at 573.15 K, the peaks of the carbons disappear, and the peak of S also almost disappears. Enlarged views of the atomic concentration profiles of the carbons of [bmim] are shown in Fig. 3c and d at the same temperature. Integration of the interfacial area shows that the concentration of C_1_ sites (which is the same as that of the [bmim] ions) decreases from 4.00 to 3.33 [bmim] ions per nm^2^ when the temperature is increased from 373.15 to 573.15 K. On the other hand, when calculating the separation between the peaks of the profiles of the C_1_ and C_2_ carbons, we can infer the orientations of the [bmim] ions at the interface. At 373.15 K, the [bmim] ions are oriented with their aliphatic chains forming a tailing angle of ∼45° with respect to the interface, pointing away from the bulk liquid, while at 573.15 K, the tailing angle becomes almost perpendicular to the interface. This amphiphilic behavior has been previously found in simulations of ionic liquids using cations [bmim] and other anions using *r*_ci_ = 10.5 Å.^4^ Silicon oxide coated on alkyl, alcoholic, and fluoroalkane chains show similar dependencies of their tailing angles when external forces are introduced.^44,45^

**Fig. 4:**
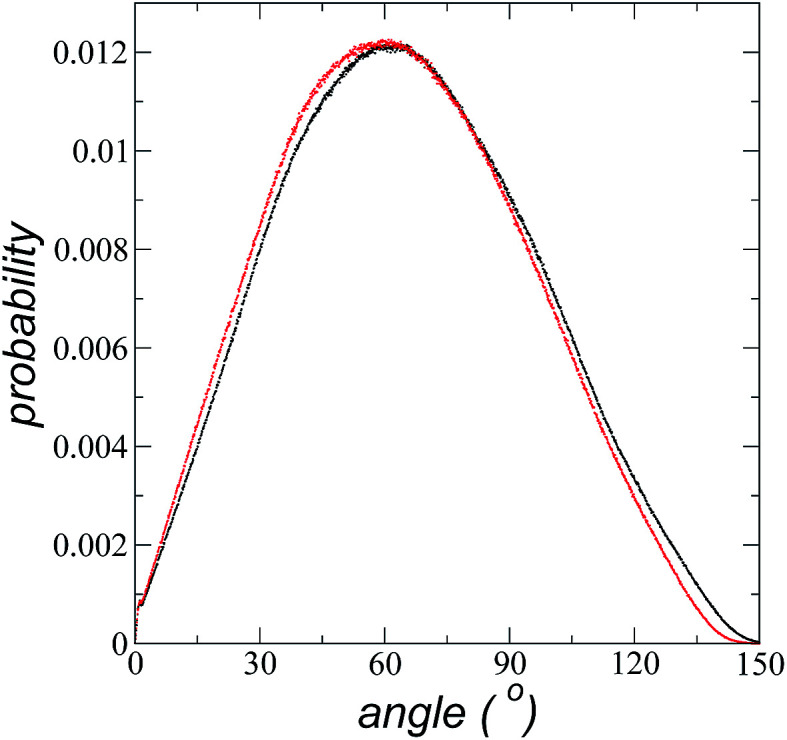
Probability distribution of the angles between the planes of the imidazolium ring of the [bmim] cation and the interface liquid/vacuum plane. Black and red dots represent distributions at 373.15 and 573.15 K, respectively.

The imidazolium ring of the [bmim] cation is planar, and the angle between the imidazolium ring plane and the interfacial plane showed a distribution of angles of around 60° in a range between 0 and 150° (Fig. 4). Temperature does not have a considerable effect on the distribution of the angles; the mean value changes by only a few degrees when the temperature is increased from 373.15 to 573.15 K, which is probably due to the structuration of the liquid phase. The distribution of the angles is consistent with the conformation that the alkyl chains take in the interface; however, a direct correlation cannot be obtained because the angle ‘imidazolium ring-N–C’ at the junction between the imidazolium ring and the alkyl group can rotate at the cost of a small energetic barrier.

### Cutoff radius effects on pressure profiles

**Fig. 5:**
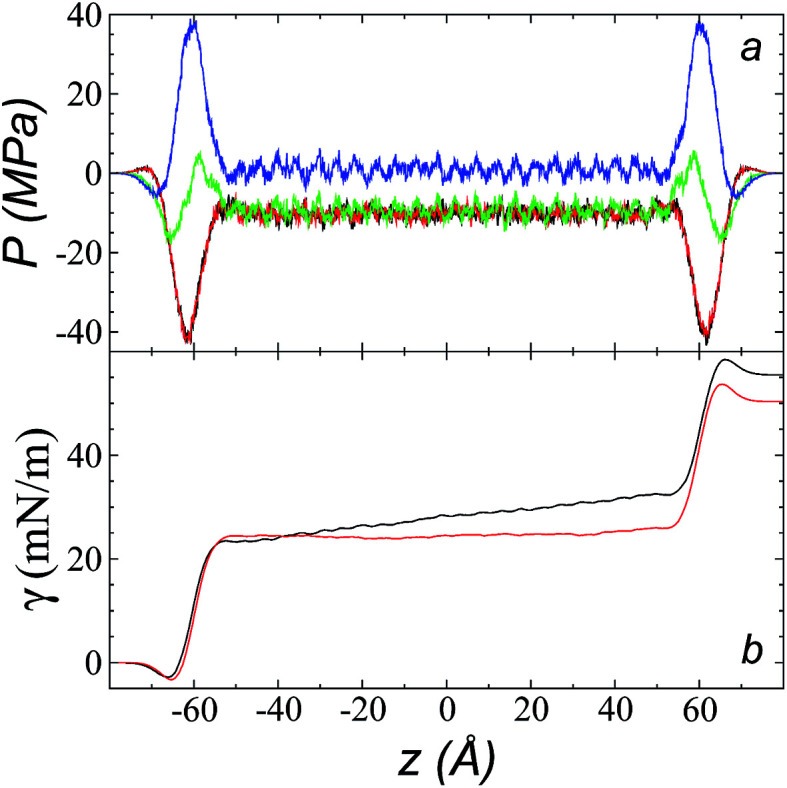
(a) Tangential (black and red) and normal (green) pressure profiles, and the difference between the normal pressure profile and average tangential profiles (blue), for the [bmim][triflate] system at 573.15 K along the inhomogeneous axis with a cutoff radius of 10.5 Å. (b) Surface tension profiles (eqn (1)) with cutoff radii of 10.5 Å (black) and 16.5 Å (red).

The pressure profiles, calculated using eqn (1) and the original *r*_ci_ and *r*_ce_ of the potential [bmim][triflate] at 573.15 K, are shown in Fig. 5a. For these, *r*_ci_ and *r*_ce_ and *P*_T_ and *P*_N_ showed differences in the bulk liquid, which should not occur in a system in mechanical balance. The *P*_T_ values were homogeneous in the region associated with the bulk liquid (−50 to 50 Å), while *P*_N_ showed structuring. When calculating the difference between *P*_N_ and the average *P*_T_, the structuring was maintained and, more importantly, the average density along the liquid phase in bulk was not zero (an average value of zero would be expected in a system in mechanical equilibrium); however, a positive average pressure difference of 0.92 MPa (±1.73 MPa) was observed, which implies that the system was not in mechanical equilibrium in the bulk liquid. We increased *r*_ci_ and *r*_ce_ by increments of 2 Å until we reached values of 30.5 and 32 Å, respectively; we found that from a *r*_ci_ of 16.5 Å, *P*_N_ lost its structuring, and the difference between *P*_N_ and the average *P*_T_ attained an average value of zero.

The use of *r*_ci_ = 10.5 Å also affected the calculation of *γ*, not only because the Lennard-Jones interactions were truncated and their contributions to *γ* were not complete, but also because the artificial mechanical instability created in the bulk liquid caused the profile of *γ* to increase continuously in the region of the bulk liquid, which should not occur in a system in mechanical equilibrium (Fig. 5b). Starting with a *r*_ci_ of 16.5 Å, the average value of the pressure difference became zero, and the contributions to *γ* in the bulk liquid were zero (Fig. 5b).

**Fig. 6:**
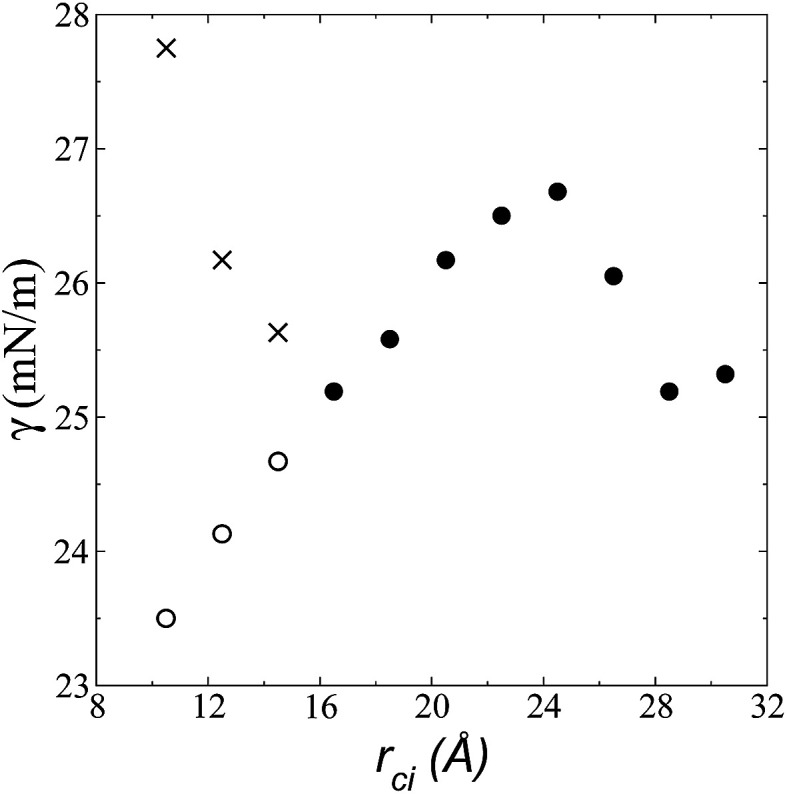
Surface tension as a function of the cutoff radius. Open circles represent values calculated using their mechanical definition, integrating the pressure difference profile only in the left interfacial region (eqn (1)), while the closed circles represent integration over the entire system. The crosses represent values calculated using the global expression (eqn (3)).

To save computational effort, *γ* is commonly calculated through the global expression3
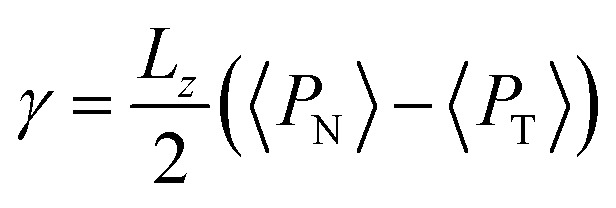
where *L*_*z*_ represents the dimension of the simulation cell along the inhomogeneous axis *z*, 〈·〉 represents temporary averages over periods on the ns scale, and the number 2 is due to the existence of 2 liquid/vacuum interfaces. In equilibrium systems, the value obtained from this global expression should be the same as that obtained by integrating the mechanical expression along the entire inhomogeneous axis (eqn (1)); however, in the global expression, it cannot be deduced whether the system is in mechanical equilibrium in the bulk liquid. Values of the global expression (eqn (3)) for the calculation of *γ* appear in Fig. 6, as well as values of *γ* using its mechanical definition (eqn (1)), integrating from −∞ to −50 Å (internal limit of the left interface) using a *r*_ci_ < 16.5 Å. Starting at *r*_ci_ = 16.5 Å, the global expression and the mechanical definition produce the same values, and only one dataset is shown. For *r*_ci_ = 10.5 Å, we observed differences in the calculation of *γ* of up to 20% between the global expression and the integration only on the left interface, without including the bulk liquid. The values obtained from integrating only on the left interface (*r*_ci_ < 16.5 Å) show a continuous profile with the values obtained from integrating on both interfaces (*r*_ci_ ≥ 16.5 Å), showing a maximum for *r*_ci_ ∼ 24.5 Å; later, the values show the beginning of what can be considered to be an oscillation zone, but with small differences between the peaks and valleys of ∼1.6 mN m^−1^. The oscillating behavior of the surface tension of simpler models of ionic liquids has been previously studied and has been associated with the size of the simulation cell, which can be indirectly associated with the cutoff radius.^46,47^

### Structuration of the bulk liquid

**Fig. 7:**
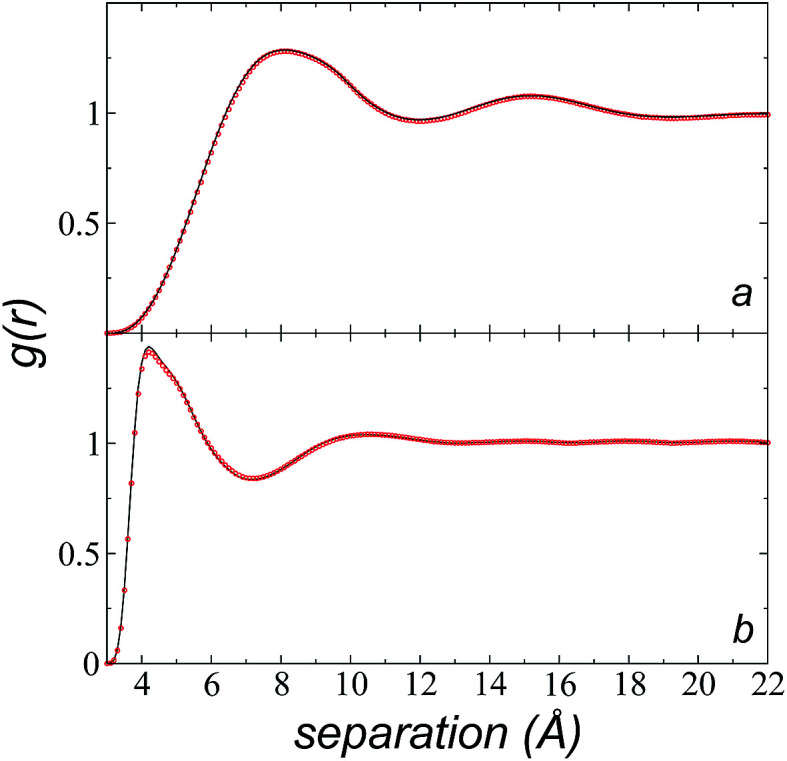
Radial distribution functions of two key sites in the [bmim] ion in the [bmim][triflate] ionic liquid at 573.15 K, which correspond to (a) one of the nitrogens of the imidazolium ring where the alkyl chain is attached, and (b) the terminal carbon of the alkyl chain, C_2_.

We calculated the radial distribution functions (RDF) of two key sites of the [bmim] ions in the bulk liquid at 573.15 K, created in the space between −50 and 50 Å in the inhomogeneous direction (Fig. 7). The RDF of one of the nitrogens in the imidazolium ring, which is the most hindered atom of the [bmim] ion, and to which the alkyl chain is attached, showed two prominent peaks at ∼8.1 and ∼15.2 Å. The second peak is larger than the cutoff radius employed in the original optimization of the potential parameters of the [bmim] and [triflate] ions; this indicates that this specific combination of ions requires a longer cutoff radius, as shown in the calculation of the pressure tensor. The RDF of the C_2_ carbon located at the end of the alkyl chain, which is the most accessible atom (excluding the hydrogens), showed one prominent peak at ∼4.2 Å and a small peak at ∼10.5 Å, which are both within the cutoff radius of the original parameterization. To verify if the larger cutoff radius employed in this work affected the results of the homogeneous liquid phase, the RDF values of the two key components were calculated using cutoff radii of *r*_ci_ = 10.5 Å and *r*_ci_ = 30.5 Å. We found that the positions of the peaks did not change, and the peaks had almost the same height; this indicates that the use of a larger cutoff radius did not affect the structural properties of the bulk liquid phase.

### Cutoff radius effects on surface tension

**Fig. 8:**
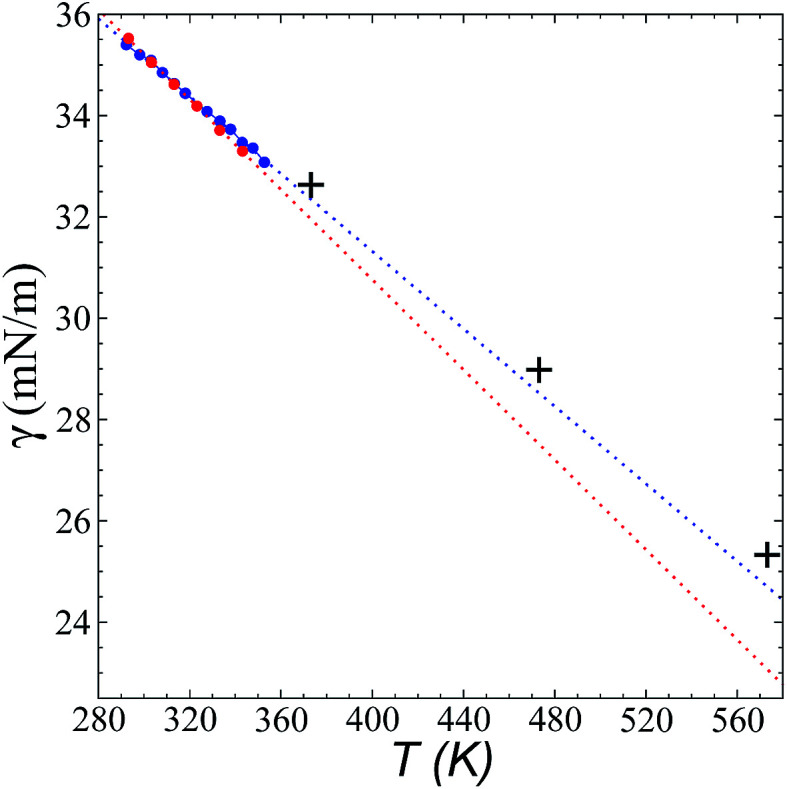
Surface tension of the [bmim][triflate] system as a function of temperature. Blue and red circles represent the experimental results of Součková *et al.*^8^ and Freire *et al.*,^9^ respectively, while the crosses represent the results of this work. Dashed lines represent linear extrapolations of the experimental data.

Fig. 8 shows the results of the calculation of *γ* at temperatures of 373.15, 473.15, and 573.15 K and *r*_ci_ = 30.5 Å, which agree well with linear extrapolations of the experimental results of Součková *et al.* at lower temperatures.^8^ Linear extrapolations of other experimental data have produced considerable deviations from our simulation results, probably due to the methods employed and the purity of the mixtures.^9^ Linear extrapolations were used in the comparisons because experimental measurements of *γ* with respect to temperature in other ionic liquids have shown good fit to the linear van der Waals–Guggenheim equation,^48^ which also describes the behavior of molten metals. At all studied temperatures, the simulations showed a maximum overestimation of ∼0.56 mN m^−1^ (573.15 K), which is equivalent to a positive deviation of ∼2.26%. This indicates that the saturated *ρ*_L_ obtained in the simulations represents an overestimation because *γ* is a strong function of the saturated *ρ*_L_. The linearity of *γ* with respect to temperature could also be assumed to be a consequence of the linearity of the saturated *ρ*_L_ that has been reported experimentally and in the simulation data acquired here. This is ultimately a consequence of the structuring of the system not only in the interface, but in the bulk liquid phase,^42,43^ although a long separation from the critical temperature may influence the linearity of this property with temperature. We also studied the properties of the system at a temperature of 298.15 K. Even though the density profiles showed a liquid phase in equilibrium, we failed to produce symmetric pressure tensors using simulation periods of up to 60 ns. At this low temperature, the liquid tends to be more structured, and we probably need more time to equilibrate the system or design a strategy to cross the local equilibrium shown by one of the interfaces.

### Contributions to surface tension

**Fig. 9:**
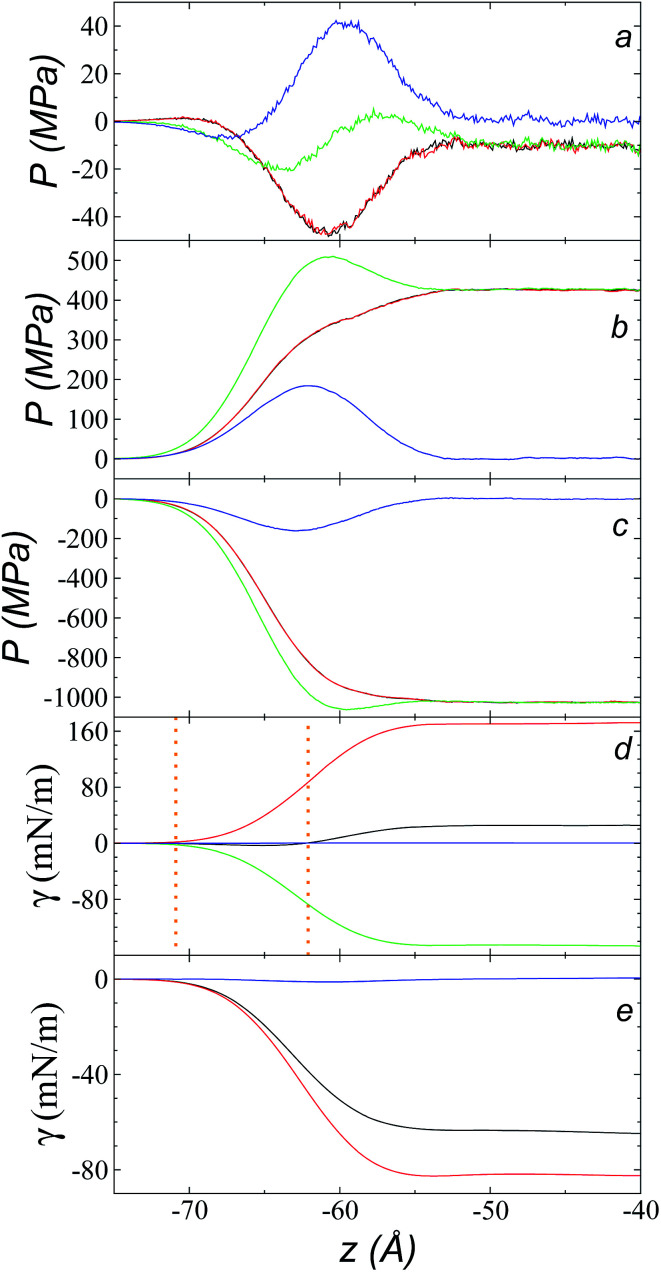
Tangential (black and red) and normal (green) pressure profiles, and the difference between the normal pressure profile and the average tangential profile (blue), for the [bmim][triflate] system at 573.15 K over the inhomogeneous axis with a cutoff radius of 30.5 Å and the contributions to the (a) total, (b) intermolecular, and (c) intramolecular pressure profiles. (d) Surface tension profiles with a cutoff radius of 30.5 Å for the total (black), intermolecular (red), intramolecular (green), and kinetic (blue) contributions. The vertical orange lines represent the positions where the different profiles begin to grow. (e) Surface tension profiles with a cut-off radius of 30.5 Å for the distance vibration (black), valence angle (red), and dihedral (blue) contributions.

Fig. 9a shows the total pressure profiles (including all intra- and intermolecular, and kinetic contributions) of *P*_N_ and the two *P*_T_ values as well as the difference between *P*_N_ and the average *P*_T_ using *r*_ci_ = 30.5 Å at 573.15 K. Using this long *r*_ci_, no structuration appeared in the bulk liquid phase, and the profile of the pressure differences in the bulk liquid showed an average value of zero (mechanical stability). Fig. 9d shows the integration profiles using the mechanical definition of *γ* (eqn (1)) for the total contributions as well as for the contributions of the inter- and intramolecular interactions, and the contribution due to the kinetic part of the pressure profile. The intermolecular contribution of *γ* shows large positive values (net contribution of ∼170 mN m^−1^). This would commonly be expected from this type of interaction, which is very dense, because the profile of differences between normal and average tangential pressures results in a profile with peaks with positive values at the interface and in the bulk liquid; however, it is interesting that the intermolecular profile, *P*_N_, at the interface showed peaks with values greater than the values of *P*_T_. The opposite would be expected (tangential intermolecular forces greater than normal forces, resulting in tension forces at the interface); thus, in the normal direction, the molecules in the interface experience stronger forces than those that occur in the bulk liquid, while the tangential forces are smaller. The fact that the pressures in all directions are positive in the regions of the bulk liquid and at the interface (Fig. 9b) indicates that, on average, the intermolecular separations are shorter than the separation corresponding to the minimum energy in the intermolecular potential (Lennard-Jones + coulombic). Thus, the intermolecular pressures are positive (repulsive), unlike what occurs in a simpler fluid, such as ethane, where the intermolecular pressures are negative (attractive) in all directions.^29^

For a simple fluid such as ethane, which is modeled as two flexibly-bonded Lennard-Jones sites, it can be concluded that the intermolecular separations are, on average, longer (attractive) than the separation corresponding to the minimum energy of the intermolecular potential (Lennard-Jones alone); however, the ions of the ionic liquid [bmim][triflate] are more complex because they also include coulombic interactions, and each interaction site has different parameters. Therefore, it is complicated to infer an average trend of how the intermolecular separations of the different sites in the interface produce this unexpected behavior.

A similar analysis of the intramolecular contributions (Fig. 9d) shows that these resulted in a negative contribution to *γ* (net contribution of about −148 mN m^−1^). The pressure profiles are negative (Fig. 9c), and they also show an inverse behavior (*P*_N_ > *P*_T_ in the interface); thus, we can infer that the molecules in the interface are in more extended states with respect to their values in equilibrium in the normal direction, which may be a consequence of what happens with the intermolecular pressures in that direction. Fig. 9e shows the *γ* profiles of the intramolecular interactions; it was observed that the main contributors to the negative value obtained are the vibration interactions of the bond distance and the valence angle. Together with the pressure profiles, we noted that the conformations of the ions in the interface have extended bonds and more open bond angles than the equilibrium values in states in which it would seem that the molecules are ‘crushed’ in the bulk liquid, as well as at the interface. This may be a consequence of the high density of the ionic liquid.

The kinetic contribution is minimal, contributing ∼0.22 mN m^−1^ to the total *γ*. The sum of all the contributions produces profiles of *P*_N_ and *P*_T_ that have physical meaning, where the tangential forces are greater than the normal forces in the interface, giving rise to the surface tension of the ionic liquid. The integration profiles of *γ* show that the inter- and intramolecular interactions begin to increase around −71 Å, while the total profile starts to increase around −62 Å; this indicates that the inter- and intramolecular contributions are almost completely canceled out in this outer interfacial region between −71 and −62 Å, which extends for ∼9 Å. As we saw in the atomic concentration profiles, this region is composed mainly of the alkyl chains of [bmim] cations and free [triflate] anions.

## Conclusions

We simulated the liquid/vacuum equilibrium of the ionic liquid [bmim][triflate] over a wide range of temperatures, from near its freezing point to 40 K below its point of thermal instability. The liquid presented linear dependencies of the density of the saturated liquid and its surface tension with respect to the temperature of the system.

The alkyl chains of the [bmim] cations were structured in, and pointing out of, the interface, with a tailing angle that became straighter as the temperature increased. Alkyl chains were highly compressed at the interface, causing an additional accumulation at the interface of a pair of [bmim][triflate] ions every 34 nm^2^ at 573.15 K. The outer region of the interface, composed of the structured alkyl chains and free [triflate] anions, did not contribute to the total surface tension; however, this region is likely to contribute significantly when pollutants or moisture accumulate within it, interacting with the alkyl chains and creating additional tension forces. The systems did not show any vaporized molecules, which may be due to this singular outer region of the interface, which is characterized as a region in balance between its intra- and intermolecular forces. Probably, only the intervention of other molecules (surfactants) or the presence of external forces would facilitate the vaporization (mobilization) of a pair of ions, creating an imbalance between the canceled forces present in that region.^49^

The use of a short internal cutoff radius (<16.5 Å) in the [bmim][triflate] system caused structuring of the bulk liquid phase in the normal direction of the interface at all simulated temperatures; additionally, it created mechanical instability due to the existence of a difference between the tangential and normal pressures in the bulk liquid, resulting in inadequate calculation of the surface tension when the global expression was used. The use of a sufficiently long cutoff radius (≥16.5 Å) for the [bmim][triflate] system avoided these problems. Any calculation of the surface tension should involve an analysis of the pressure profiles to avoid artefacts caused by a limited cutoff radius. The radial distribution functions of key sites in the [bmim] structure also show peaks at positions larger than the cutoff radius employed in the original parameterizations; this necessitates a larger cutoff radius to properly account for these characteristic regions, where the radial distribution is larger than the value distribution.

The positive contributions of the intermolecular interactions to the surface tension reached values of up to 6.8 times the value of the total surface tension. Because the liquid is a very dense system, the intermolecular pressures showed positive values (repulsive) in the bulk liquid and the interface; this presented unexpected behavior, where the normal pressures were greater than the tangential pressures. The intermolecular contributions to surface tension were compensated by negative contributions, mainly due to the intramolecular interactions of the vibrations of the bond and the angle of valence. This showed that at the interface, the molecules were more extended (crushed) than in the bulk liquid, which is probably a consequence of repulsive intermolecular separations. This may explain the almost null value of the vapor pressure, because the molecules under these normal repulsive pressures were strong and only the intermediation of the intramolecular forces kept them attached to the interface. The total pressure profiles show values that have physical meaning, where the tangential pressures were stronger than the normal pressure and were the origin of the surface tension.

## Conflicts of interest

There are no conflicts to declare.

## Supplementary Material
